# Exploring chemoselective *S*-to-*N* acyl transfer reactions in synthesis and chemical biology

**DOI:** 10.1038/ncomms15655

**Published:** 2017-05-24

**Authors:** Helen M. Burke, Lauren McSweeney, Eoin M. Scanlan

**Affiliations:** 1 School of Chemistry, Trinity College Dublin, Dublin D2, Ireland

## Abstract

*S*-to-*N* acyl transfer is a high-yielding chemoselective process for amide bond formation. It is widely utilized by chemists for synthetic applications, including peptide and protein synthesis, chemical modification of proteins, protein-protein ligation and the development of probes and molecular machines. Recent advances in our understanding of *S*-to-*N* acyl transfer processes in biology and innovations in methodology for thioester formation and desulfurization, together with an extension of the size of cyclic transition states, have expanded the boundaries of this process well beyond peptide ligation. As the field develops, this chemistry will play a central role in our molecular understanding of Biology.

## Abstract (web summary)

The conversion of thioesters to amides via acyl transfer has become one of the most important synthetic techniques for the chemical synthesis and modification of proteins. This review discusses this S-to-N acyl transfer process, and highlights some of the key applications across chemistry and biology.

Chemoselectivity refers to the preferential reaction of a single chemical reagent with one of two or more different functional groups^1^. One of the most formidable challenges presented to synthetic chemists involves achieving high levels of chemoselectivity and regioselectivity amongst the myriad of reactive functionalities present in biological systems. Despite the onerous challenges, chemists continue to discover and develop robust and efficient chemical ligation methodologies that enable exquisitely high levels of selectivity^2^,^3^,^4^. The chemoselective formation of amide bonds is a critical reaction in biology for protein synthesis and post-translational modification of proteins (PTMs), it is also widely utilized in organic synthesis and medicinal chemistry^5^,^6^,^7^. Amide bond formation requires chemoselective ligations that can furnish specific amide products in the presence of functional groups such as unprotected amines, carboxylic acids and alcohols^6^,^7^. The chemoselectivity of the *S*-to-*N* acyl transfer process arises from the unique reactivity of thioesters that renders them ideal intermediates for acyl transfer processes. Thioesters have been found to be more reactive then the corresponding oxoesters towards most nucleophiles (with the notable exception of hydroxide and alkoxides). The enhanced reactivity of thioesters may be due to a poor C-S π overlap that lowers the overall thermodynamic stability of the thioester relative to the oxoester^8^,^9^,^10^. Thioesters have been exploited as reactive intermediates by chemists to perform sophisticated ligation reactions under neutral conditions in an aqueous environment and have inspired a recent surge in the development of synthetic and biosynthetic ligation methodologies^11^,^12^,^13^,^14^,^15^. Efficient cascade processes involving thioester formation and subsequent acyl transfer to N-, O- or Se- groups are at the core of several critical biological processes^14^. For example, Acetoacetyl-CoA functions as an efficient acyl transfer reagent in the Krebs cycle^16^. Thioesters are also key intermediates in protein ubiquitination^17^,^18^, intein splicing^19^,^20^ and the covalent modification of bacterial cell-surface proteins^21^,^22^. Thioesters are formed in transglutamination and in glutathione (GSH) biosynthesis. The abundance of thioester mediated processes in nature has led to speculation regarding the role of these derivatives in the origin of life^23^. In this review we will discuss the key role of *S*-to-*N* acyl transfer in biological systems and recent developments and applications of *S*-to-*N* acyl transfer in chemical synthesis (semi-synthesis), chemical biology, supramolecular chemistry and chemical probes. Both inter- and intramolecular acyl transfer reactions will be discussed.

## *S*-to-*N* acyl transfer reactions in biology

The *S*-to-*N* acyl transfer presents an efficient chemoselective ligation process utilized by nature to synthesize amide bonds. Among the most heavily investigated biological processes involving *S*-to-*N* acyl transfer are protein ubiquitination, intein-mediated protein splicing, sortase mediated protein modification and transglutamination.

## Ubiquitination

**Figure 1:**
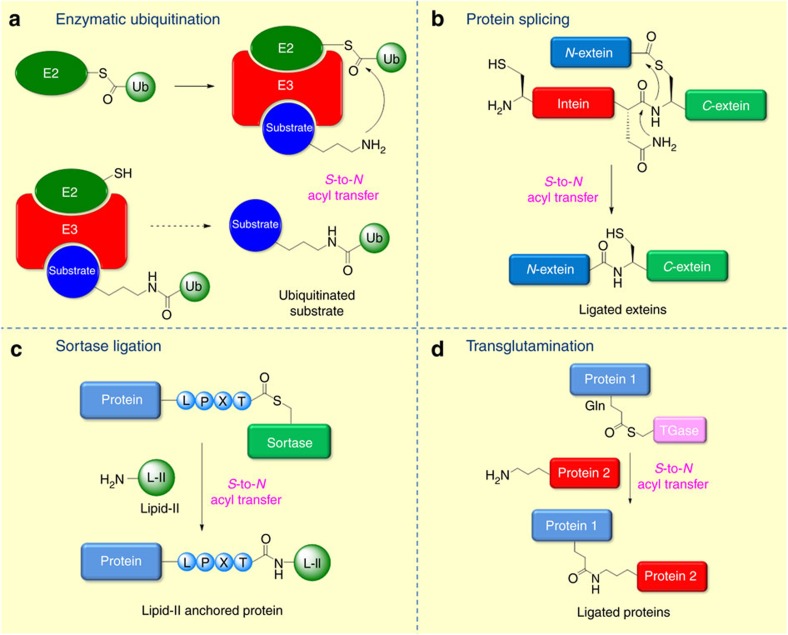
*S*-to-*N* acyl transfer reactions utilized in nature. (**a**) Enzymatic ubiquitination where *S*-to-*N* acyl transfer forges an isopeptide bond between a terminal glycine and a lysine residue (**b**) protein splicing where *S*-to-*N* acyl transfer ligates the extein fragments (**c**) sortase mediated ligation where *S*-to-*N* acyl transfer introduces isopeptide bond between Thr residue of protein and Lys of lipid-II. (**d**) Transglutamination where *S*-to-*N* acyl transfer ligates proteins via an isopeptide bond.

Ubiquitination is an important post-translational modification implicated in several cellular pathways such as protein degradation, inflammatory responses and DNA repair^24^,^25^,^26^. The process of ubiquitination involves formation of an isopeptide bond, a non-canonical amide linkage, between the *C*-terminal Gly residue of Ubiquitin (Ub) and the ɛ-NH_2_ of the substrate Lys. Isopeptide bonds formed through lysine residues mediate several critical biological processes through intermolecular cross-linking of proteins^27^. Ubiquitination is a multi-step process orchestrated by three key enzymes: activating enzyme (E1), conjugating enzyme (E2), and ligase (E3), with the overall process depicted in Fig. 1a (ref. 17). The first step is an ATP-dependent process which involves E1 forming a thioester linkage between a catalytic Cys residue and the Ub *C*-terminal Gly residue. In the second step, the activated Ub molecule is then transferred to an E2 enzyme via a *trans*-thiolation process with an E2 Cys residue. In the third step, the E3 ligase catalyses the formation of the isopeptide bond between the Ub C-terminal Gly and the ɛ-amino of the substrate Lys residue through a chemoselective *S*-to-*N* acyl transfer^28^,^29^,^30^. There are three classes of E3 ligases (i) RING E3s, which bind to the E2-Ub thioester and substrate and catalyse attack of the substrate lysine on the thioester (ii) HECT and (iii) RBR E3s, which both have active site cysteines and catalyse substrate ubiquitination in a two-step process involving formation of a thioester with the E3 followed by attack of the substrate lysine to form the isopeptide bond. Although the molecular detail of ubiquitination is unknown, it is postulated that RING E3 functions in positioning the reactive thioester of E2 and the ubiquitin C-terminus in the correct orientation to react with the attacking lysine of the substrate. Conserved residues in the active site function to catalyse the *S*-to-*N* acyl transfer step^17^. The conjugation of Ub to a protein may involve the addition of a monomer (monoubiquitination) or as a chain of Ubs of various types (polyubiquitination) with these different modifications producing a variety of molecular signals depending on the nature of the ubiquitination^31^. Recently it has been reported that the ubiquitin-conjugating enzyme E2(Ube2w) employs a novel mechanism to enable specific ubiquitination of the α-amino group of its substrates. This process involves recognition of backbone atoms of intrinsically disordered *N*-termini^32^. Post-translational modification of proteins by the small ubiquitin-related modifier (SUMO) protein proceeds through a ubiquitin-like pathway also involving a critical *S*-to-*N* acyl transfer step^33^.

## Protein splicing

Protein splicing is an intramolecular reaction of a protein whereby an internal protein segment (intein) is excised from a protein with concomitant ligation of the adjacent *C*-terminal and *N*-terminal external proteins (exteins). An intein mediates its own excision from a peptide sequence through a series of acyl transfer reactions^20^,^34^. The first step of protein splicing involves an *N*-to-*S* acyl transfer between the intein Cys and *N*-extein producing a thioester intermediate (the *N*-to-*O* acyl transfer process involving a Ser residue is also well known for inteins but for the scope of this review we will only consider the Cys mediated process). Although this rearrangement appears to be thermodynamically unfavourable, changes in the molecular architecture of the intein push the equilibrium towards thioester formation^35^. (The reversibility of the *S*-to-*N* /*N*-to-*S* acyl transfer is discussed in detail in Box 1). A subsequent *trans*-thioesterification step transfers the *N*-extein to the *C*-extein via a Cys residue. Cyclisation of a conserved asparagine (Asn) residue forming a succinimide intermediate and subsequent *S*-to-*N* acyl transfer forms the new peptide bond, completing the protein splicing process. Inteins have been widely exploited for the chemical synthesis of proteins through the formation of reactive thioesters on expressed proteins^20^.

## Sortase transpeptidase enzymes

Sortases are transpeptidase enzymes that covalently modify cell surface proteins of bacteria. They are responsible for the surface display and cell-wall anchoring of proteins encoding the sortase recognition motif LPXTG^22^. They are classified into four groups (sortase A–D) based on sequence homology, the substrate for sortase cleavage and the nucleophile that reacts with sortase thioester. Sortase class A enzymes, found in all Gram-positive bacteria are commonly referred to as housekeeping sortases. They recognize the amino acid (AA) sequence LPXTG at the carboxyl terminus of surface protein precursors. The products of sortase class A reactions are surface proteins that are covalently linked to lipids and subsequently incorporated into the cell-wall envelope^36^. Sortase class B enzymes recognize a unique NP(Q/K)TN sorting signal in proteins and are involved in haem-iron scavenging, and in cross-linking anchored haem-containing products near membrane transporters. The class C sortases polymerize pili by catalysing transpeptidation reactions forming covalent bonds between individual pili subunits. Sortase class D enzymes are expressed in a variety of spore-forming microorganisms; they function by recognizing and cleaving the sorting signals of selected substrates to immobilize anchored products in the cell wall envelope during spore formation. All classes of sortases recognize a specific sorting signal at the C terminus of their target protein. The specificity of cleavage is determined by recognition of sortase-specific sorting signals; each sortase cleaves its specific sorting signal and subsequently forms a thioester bond between the sortase active site and a residue in the sorting signal. This thioester intermediate then undergoes a chemoselective *S*-to-*N* acyl transfer onto a specific nucleophilic amino acid, thus specifying the cell wall component to which the protein becomes ligated^22^.

## Transglutamination

Transglutaminase enzymes (TGs) are transferases that catalyse the calcium dependant formation of inter- or intramolecular isopeptide bonds between proteins by crosslinking protein bound glutamine and lysine amino acids^37^. TGs are homologous to the papain family of proteases with which they share significant active site sequence and structural similarities^38^. During transglutamination, a glutamine side chain on a substrate protein is attacked by the active site Cys of a transglutaminase enzyme with formation of a thioester intermediate. The TG then mediates *S*-to-*N* acyl transfer to a Lys residue on a second substrate molecule with formation of an isopeptide bond. TGs exhibit remarkable specificities not only for the Gln site of hydrolysis, but also for the Lys residue of proteins with which they react. Recent studies suggest that the specificity of Lys residues TGs is not encoded in the primary amino acid sequence surrounding the target Lys but is primarily dependant on its positioning in an accessible segment of the protein structure^38^.

## Glutathione biosynthesis

The first step in glutathione (GSH) biosynthesis is formation of γ-glutamyl cysteine by the enzyme glutamate-cysteine ligase (GshA), however, in the case of bacteria lacking GshA it has been proposed that formation of γ-glutamyl cysteine occurs via an *S*-to-*N* acyl transfer pathway involving the selective interception of ProB-bound γ-glutamyl phosphate by cysteine residues^39^.

These biological examples of chemoselective amide bond formation clearly demonstrate the synthetic potential of the *S*-to-*N* acyl transfer process. These processes are multistep and involve the formation of a suitably labile thioester, followed by correct positioning of both the thioester and amine nucleophile in the active site. Ligation reactions are typically catalysed by AA residues located at the active site^40^. Not only do these biological processes highlight the synthetic potential of the *S*-to-*N* acyl transfer, they also serve as inspiration for synthetic molecular processes utilizing *S*-to-*N* acyl transfer as a key step. A key requirement for efficient *S*-to-*N* acyl transfer is access to a suitable thioester and a number of elegant methods for their synthesis via synthetic and biological approaches have been reported, including pushing the *S*-to-*N* acyl transfer process into reverse (Box 1)^13^,^41^,^42^,^43^. A common synthetic method employed for the preparation of thioesters involves coupling of the C-terminus of a protected peptide/glycopeptide with benzylthiol using PyBOP/DIPEA as coupling reagents (to avoid racemization)^44^. Wong and co-workers^45^ have reported solid-phase synthesis of peptide and glycopeptide thioesters using a side chain anchoring strategy. In this approach, the C terminus is coupled with either a thiol or a thioester AA residue to furnish the peptide thioester which is subsequently released from the solid phase^45^. Dawson and co-workers^46^ have reported the synthesis of thioesters through formation of a C-terminal *N*-acylurea moiety. Following SPPS an aminoanilide undergoes specific acylation and cyclization to furnish the resin-bound acylurea peptide. Following cleavage from the resin and global deprotection, the peptide *N*-acylurea can undergo thiolysis to yield the peptide thioester^46^. Liu and co-workers^47^ have reported the use of peptide hydrazides as thioester surrogates in NCL. The peptide hydrazides are prepared through SPPS and subsequent NaNO_2_ activation and thiolysis on treatment with 4-mercaptophenylacetic acid (MPAA), furnishes a peptide thioester^47^. Recently Chatterjee and co-workers have reported the application of an *N*-mercaptoethoxyglycinamide (MEGA) solid-phase linker for the facile synthesis of peptide α-thioesters. Thioester formation occurs from the *N*-oxyamide via an *N*-to-*S* acyl shift through a 6-membered cyclic intermediate^48^. Several other efficient methods for thioester synthesis have been developed.

## Synthetic applications of *S*-to-*N* acyl transfer

A broad range of synthetic applications have been developed that utilize the *S*-to-*N* acyl transfer as a key step to introduce an amide bond. Particular focus has been placed on the synthesis of peptide bonds through Native Chemical Ligation (NCL) and related methodologies.

**Figure 2:**
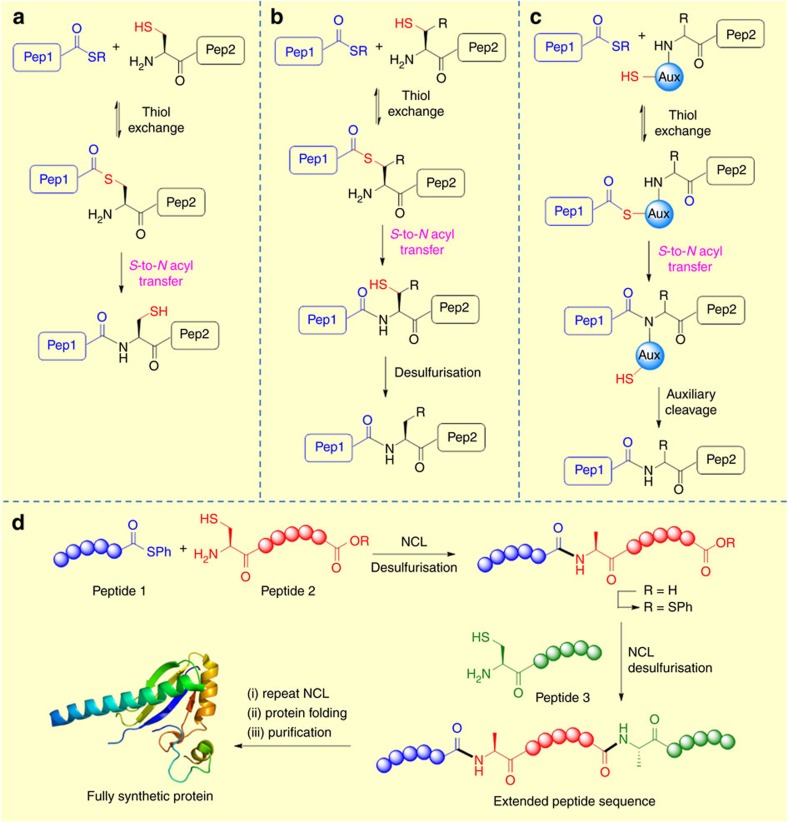
Mechanisms of synthetic amide-forming reactions involving S-to-N acyl transfer. (**a**) Native Chemical Ligation (NCL) (**b**) Ligation-desulfurization (**c**) Auxiliary-mediated ligation (AML) (**d**) Sequential NCL applied to chemical protein synthesis.

The most heavily investigated and widely utilized synthetic application of *S*-to-*N* acyl transfer is undoubtedly native chemical ligation. NCL was discovered by Kent and co-workers as an efficient methodology for peptide ligation^49^. The process gained widespread attention with the reported synthesis of human interleukin-8 (IL-8) by Kent and co-workers^50^, a major synthetic achievement which served to highlight the synthetic potential of NCL for chemical protein synthesis. The reaction involves an *N*-terminal cysteine and a *C*-terminal thioester reacting in a reversible *trans*-thioesterification step to form a thioester intermediate. This intermediate can subsequently rearrange via an intramolecular *S*-to-*N* acyl transfer through a 5-membered transition state, to furnish a native peptide bond at the ligation site (Fig. 2). NCL has revolutionized the total chemical synthesis of peptides and proteins as it enables the chemoselective ligation of two mutually reactive peptide entities in aqueous solution at neutral pH to form a single product in near-quantitative yields. Accordingly, NCL has become one of the most commonly employed methods for peptide ligation. Following a number of substrate studies, the steric nature of the *C*-terminal thioester was found to be the most influential factor in NCL. Ligations occur faster at less hindered amino acids, such as Gly or Ala. Dawson and co-workers explored the scope of the NCL process and demonstrated that ligations were possible using all 20 amino acids, however, ligations sites involving valine (Val), isoleucine (Ile), and proline (Pro) were less favourable due to slower ligation rates^51^. Alkyl thioesters can easily be prepared by SPPS but are relatively unreactive in NCL, with time periods of 24–48 h required for the *trans*-thioesterification step to reach completion^49^. The initial rate-determining *trans*-thioesterification step can be accelerated through the addition of an exogenous aryl thiol. Generally, aryl thioesters are more reactive than alkyl thioesters due to their lower pKa values which make them better leaving groups, therefore, addition of an aryl thiol can promote the *in situ* formation of a more reactive thioester thus accelerating the rate of ligation^52^,^53^. Payne and co-workers^54^ have demonstrated that trifluoroethanethiol TFET can be efficiently employed as an additive in NCL to enable ligations with rates comparable to those obtained with aryl thioesters. This reagent was demonstrated to be efficient in ligation−desulfurization chemistry for protein synthesis without the requirement for intermediate purification or removal/capture from the reaction mixture.

The broad application of NCL has elevated it to a privileged position among chemical methods for peptide, protein and glycopeptide synthesis and it serves to highlight the synthetic potential of *S*-to-*N* acyl transfer in chemoselective ligations. Some spectacular synthetic achievements have been reported, in particular for the chemical synthesis of proteins (see applications of NCL for the synthesis of proteins). Using SPPS to assemble the two peptide entities, NCL was used in a convergent manner to allow access to peptides and proteins that were previously inaccessible by chemical synthesis approaches, with the synthesis of a HIV-1 protease covalent dimer being one of the most impressive examples reported to date^55^. This example, reported by Kent and co-workers^55^, is a 203-residue polypeptide and is one of the largest linear polypeptides prepared by chemical synthesis.

Despite its utility, certain limitations of NCL have persisted. An ongoing criticism of NCL is the requirement for an *N*-terminal Cys at the ligation site. This limitation is coupled with the fact that Cys is not a very abundant amino acid in nature (∼1.7% in human proteins) and is seldom suitably distributed throughout a peptide sequence so as to facilitate NCL^11^,^13^. Furthermore, NCL in its original form is not fully convergent with multiple peptide fragments being joined in a linear fashion from the C-terminus to the *N*-terminus. However, numerous synthetic advancements have been reported to address these limitations, including desulfurization and Auxiliary Mediated Ligation (AML), Fig. 2.

**Figure 3:**
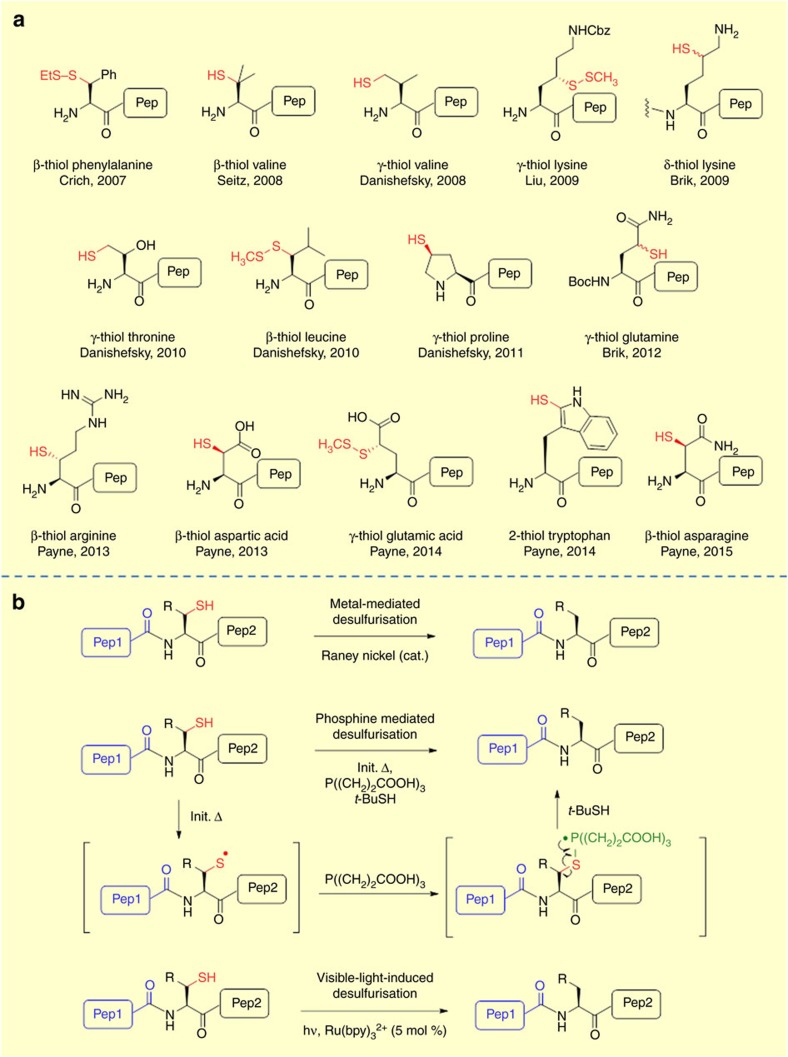
Native Chemical Ligation-Desulfurization. (**a**) Structures of thiol containing amino acids investigated for NCL-desulfurization (**b**) Methods for desulfurization of thiol groups following NCL.

One of the most important synthetic advancements in overcoming the requirement for Cys at the ligation site was first reported by Dawson and co-workers and involves the use of NCL coupled with desulfurization^56^. This concept was initially proved using Ala residues where a Cys residue was mutated to Ala after NCL. Desulfurization from Cys to Ala was promoted using Raney nickel as a reagent. This allowed for ligation at Xaa–Ala sites which are naturally more abundant than Cys. Inspired by this initial work, several groups investigated and extended this strategy to other Cys-surrogates, in the form of mercaptoamino acids whereby a thiol group is incorporated into amino acids, making ligation at Phe, Lys, and Val possible, for example, refs 57, 58, 59. Indeed, it is now possible to perform ligations at 15 out of the 20 natural amino acids^60^,^61^. Figure 3 shows the structures of a range of thiol containing amino acids that have been prepared and investigated for NCL. Generally, mercaptoamino acids are built off the side-chains to preserve stereochemistry at the alpha carbon. The synthesis of mercaptoamino acids typically involves installation of the key thiol auxiliary unit into a natural amino acid and usually requires a multistep synthesis^61^. Only the β-thiol valine (penicillimine) and γ-thiol proline derivatives are commercially available. Several impressive total-syntheses of proteins were achieved through the ligation–desulfurization approach, however, certain limitations of this methodology also exist. The method requires access to specific thiol-modified amino acids for every ligation junction. The limited commercial availability of thiolated amino acid building blocks presents a challenge for their general application in protein synthesis. Although the desulfurization strategy reported by Dawson represented a major milestone in the use of NCL at non-Cys ligation sites, problems such as low product recoveries and non-selective reduction of thioesters and thioethers led to the development of free-radical desulfurization methods. Hoffman and co-workers^62^ were the first to disclose a desulfurization reaction between thiol and trialkylphosphite derivatives under thermal and photochemical conditions. Shortly thereafter, Walling and Rabinowitz^63^ proposed the mechanism of the desulfurization. It was proposed that a reactive alkylthiyl radical adds to the phosphite generating a phosphoranyl radical intermediate which can subsequently eliminate to an alkyl radical. Hydrogen abstraction from the parent thiol can furnish the alkane product while also propagating the chain reaction. This approach was later modified by Danishefsky and co-workers resulting in a more environmentally friendly dethiylation method for use in NCL^64^. They used radical initiator 2,2′-azobis[2-(2-imidazolin-2-yl)propane]dihydrochloride (VA-044) and tris(2-carboxyethyl)phosphine) (TCEP), a widely used disulfide reducing agent in NCL, as the phosphine source. On heating in aqueous solution, the initiator decomposes to give 2-isopropyl-4,5-dihydro-1H-imidazole radical initiator which on reacting with Cys generates a thiol radical. The thiol radical can then react to form a phosphine radical intermediate which then undergoes C-S bond scission to generate an alkyl radical which can subsequently be quenched by addition of a thiol additive, such as t-butyl mercaptan (*t*-BuSH), through hydrogen abstraction^11^. This mild process furnished the desired products with yields in excess of 80% and the method was compatible with a variety of functionalities such as methionine, thioesters, and thiazolidine protected Cys residues. Recently, Guo and co-workers have reported a photocatalytic approach to desulfurization employing a ruthenium catalyst in the presence of a phosphine reagent. The methodology was successfully applied to peptide, cyclic peptide and glycopeptide synthesis^65^. The use of visible light offers advantages over UV initiated methods since it is known that exposure to UV can damage protein structures^66^.

### Auxiliary mediated ligation

**Figure 4:**
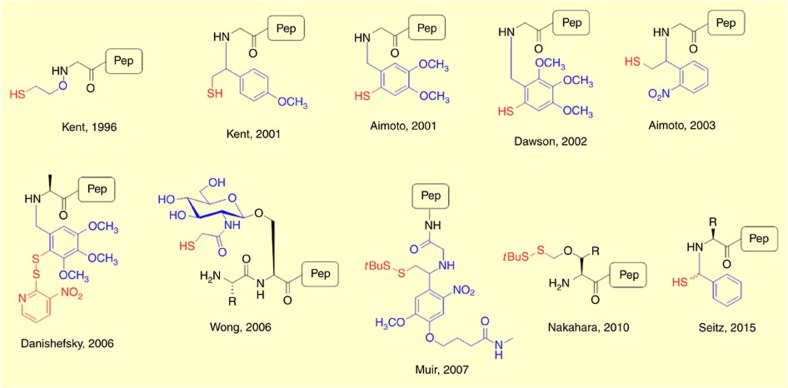
Auxiliary Mediated Ligation (AML). Structures of common auxiliaries investigated for AML.

An alternative approach to overcome the requirement of Cys for peptide ligation is auxiliary mediated ligation (AML), where an auxiliary-modified peptide is used directly in NCL (Fig. 2c). The auxiliary should facilitate *S*-to-*N* acyl transfer to form the native peptide bond and then be removed quantitatively under mild conditions. A wide range of auxiliaries with varying cleavage conditions have been developed. In early examples, the α-amino group of the peptide was used to anchor the thiol auxiliary but this approach often results in low conversion due to steric hindrance and has been mainly limited to ligations containing at least one Gly residue. A more general approach involves anchoring the auxiliary onto a side-chain functional group which is subsequently converted back into the native AA following ligation. Using this approach, the ligation has been reported to proceed efficiently via *S*-to-*N* acyl transfer through five-, six- or even larger-membered cyclic transition states. Examples of commonly utilized auxiliaries developed are shown in Fig. 4. Auxiliary approaches are usually built off Gly because of the lack of stereochemistry and simplicity of the submonomer approach for synthesis of the relevant peptoids.

The earliest examples of auxiliary mediated ligation were reported by Kent and co-workers^67^,^68^ with the oxyalkyl auxiliary on the amide bond removed by treatment with Zn under acidic conditions to give a native peptide bond. Danishefsky and co-workers developed a cysteine-free NCL approach suitable for the synthesis of *N*- and *O*-linked glycopeptides, inspired by the auxiliary methodology reported by Dawson^69^,^70^. Aimoto and co-workers reported a mild, photocleavable auxiliary compatible with polypeptide synthesis^71^. Sugar assisted ligation (SAL), is a form of AML introduced and developed by the Wong group^72^,^73^. The thiosugar auxiliary is prepared as a glycosyl amino acid building block and installed during SPPS near the ligation site, where it mediates amide bond formation through an initial thiol-exchange with a peptidyl thioester. The thiol auxiliary can be subsequently removed using established desulfurization methods^56^. A key difference between SAL and conventional NCL is the size of the cyclic transition state with SAL proceeding via a 14-membered transition state (a detailed discussion on the size of cyclic transition states in *S*-to-*N* acyl transfer is provided in Box 2). Consequently, SAL reactions tend to be slower when compared with NCL which proceeds through a 5- or 6-membered cyclic transition state^74^,^75^. However, the large transition state in SAL is suggested to be facilitated by the sugar moiety which acts as a rigid scaffold for the reacting groups. As with NCL, the steric nature of the thioester component is the most influential factor in SAL efficiency with residues containing Pro, Val, Iso, Leu, and Thr known to be particularly difficult^51^,^74^. To achieve ligation efficiency, it was found that the ligation site must include at least one Gly which may limit the general applicability of SAL in glycopeptide synthesis^72^. In a related approach, Brik and co-workers reported the application of a modified cyclohexane auxiliary as a scaffold to mimic the role of the glycan in SAL and promote efficient ligation^76^. Muir and co-workers reported a photocleavable auxiliary suitable for protein ubiquitination. The auxiliary handle was used to direct site-specific ubiquitylation at one of three Lys residues in the target protein^77^. Seitz and co-workers have recently reported an auxiliary that enables ligation at sterically demanding junctions. The auxiliary can be cleaved under mild basic conditions and is compatible with SPPS^78^. It is clear from these and other studies that the auxiliary mediated approach offers a useful extension of NCL and can be applied to the total synthesis of complex functionalized proteins.

### Kinetically controlled ligation and sequential NCL

The issue of the lack of convergence in NCL was tackled by Kent and co-workers^79^ who reported Kinetically Controlled Ligation (KCL). KCL exploits the difference in reactivity between alkyl and aryl thioesters, allowing the pre-organisation of the order of NCL reactions. By conducting a one-pot experiment, they demonstrated that the more reactive aryl peptide thioester is ligated with a Cys peptide bearing a less reactive alkyl thioester with the alkyl thioesters remaining intact until further activation^80^. This approach allows for selective peptide elongation in both the *N*- and *C*-terminal direction. The utility of KCL was demonstrated through the synthesis of crambin, a 46-amino acid protein, by a fully convergent synthesis involving six peptide segments and only two protecting groups^81^. KCL was explored in greater detail by Lee and co-workers^82^. They demonstrated that optimal KCL can be achieved through combination of a reactive AA such as Gly or Ala with a less reactive AA such as Val. As discussed previously, fluorinated alkyl thiol additives have been used to enable one-pot ligation-desulfurization chemistry for efficient protein synthesis^54^. The reactivity of the fluorinated alkyl thioester was found to be comparable to that of the aromatic derivative, rendering it extremely useful for KCL. In a recent innovation, Payne and co-workers have dramatically reduced the time required for NCL through the reaction between peptide selenoesters and peptide dimers bearing *N*-terminal selenocystine. The process proceeds in aqueous buffer to afford native amide bonds without the requirement of additives. The resulting selenocysteine can be deselenized to afford alanine residue at the ligation site^83^.

**Figure 5:**
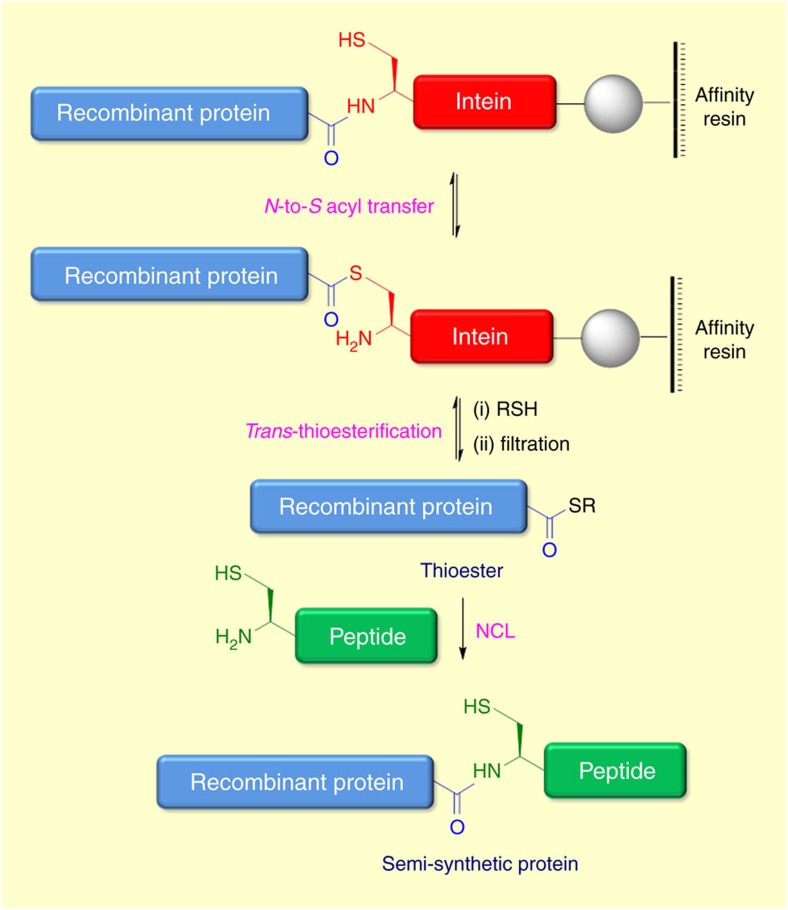
Expressed protein ligation (EPL) for the synthesis of proteins. Initial *N*-to-*S* acyl transfer generates a thioester which subsequently undergoes *trans*-thioesterification with an exogenous thiol. The resulting recombinant protein thioester can participate in NCL with synthetic cysteinyl peptides.

The desire to combine recombinant proteins with established NCL methods led to the development of expressed protein ligation (EPL) by Muir and co-workers in 1998 which is based on the natural occurring protein splicing mediated by inteins^84^. EPL vastly expands the size limitation and scope of protein synthesis by combining NCL with recombinant protein production. This can be accomplished through ligation of a recombinant protein thioester with a synthetic peptide containing an *N*-terminal Cys (Fig. 5). The protein of interest is expressed *N*-terminally to an intein domain which is bound through its *C*-terminus to a solid support. The intein is modified through mutation of the conserved Asn residue halting *C*-terminal cleavage and splicing. An initial *N*-to-*S* acyl transfer generates a thioester intermediate which undergoes *trans*-thioesterification with an exogenous thiol. Subsequent filtration releases the desired recombinant protein thioester which can then participate in NCL with synthetic cysteinyl peptides^34^,^85^. The accessibility of recombinant products of hybrid biological and chemical origin has enabled EPL to emerge as a powerful tool in protein engineering through the introduction of unnatural amino acid sequences, various PTMs, fluorophores, or reactive synthetic handles allowing for further modification or surface immobilization^86^,^87^,^88^,^89^. The applicability of EPL is well illustrated in the production of fluorescent enzyme biosensors. An example reported by Muir and co-workers demonstrated the incorporation of two fluorophores within Crk-II, a cAbl protein tyrosine kinase substrate involved in several signalling pathways, using EPL. This enabled real-time monitoring of protein phosphorylation and kinase activity, using fluorescence resonance energy transfer (FRET) measurements^90^. Recently butelase mediated ligations have been reported by Tam and co-workers as an alternative to EPL^91^. Butelase 1 is a peptide ligase utilized for high-efficiency cyclization and ligation of peptides and proteins ranging in size from 10 to >200 residues. Both inter- and intramolecular ligation pathways proceed via an *S*-to-*N* acyl transfer mediated by a butelase thioester intermediate. Sortase mediated ligations have been employed for the site-specific modification of proteins with fluorophores, biotin, proteins or lipids. This approach involves engineering of target protein with a sortase-recognition motif (LPLPXTG) at the site where modification is desired. Subsequent treatment with sortase cleaves the protein between the threonine and glycine residues, facilitating the attachment of an exogenously added oligoglycine peptide modified with the functional group of choice^21^.

**Figure 6:**
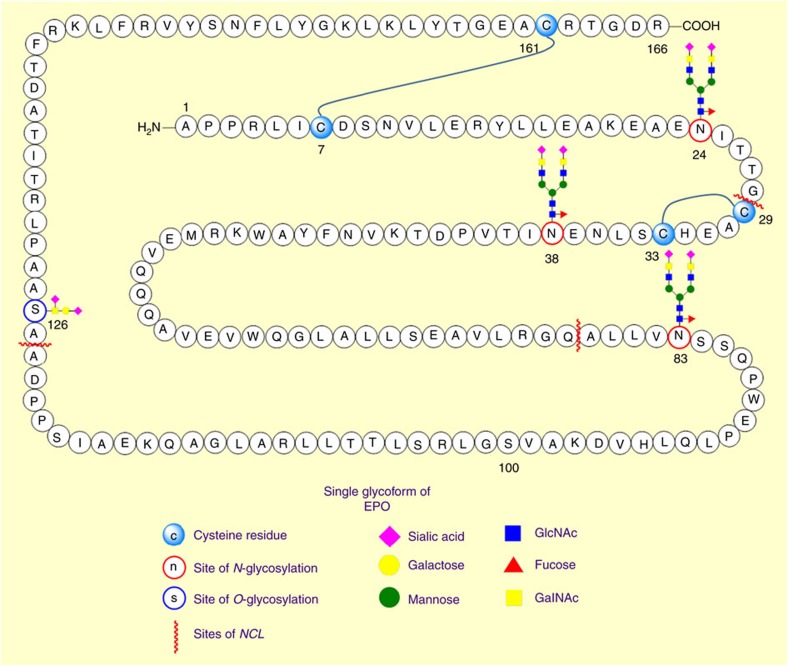
Single glycoform of EPO. Schematic representation of synthetic homogeneous EPO glycoform. *N*-glycosylation sites are shown at position 24, 38 and 83. *O*-glycosylation is shown at position 126.

NCL, in combination with related cysteine-free ligation methods and EPL, represents one of the most convenient and popular methods for the synthesis of peptides and proteins of biological and therapeutic interest. The synthetic potential of the methodology is abundantly clear from the numerous reported total-syntheses of proteins that utilize this approach. An impressive array of complex peptides and proteins have been successfully synthesized by NCL including the sialic acid-binding lectin siglec-7 (127 AA)^92^, human interleukin-6 glyoprotein (183 AA)^93^, and the pore-forming antimicrobial protein caenopore-5 (82 AA)^94^. In a ground-breaking study, Valiyaveetil and co-workers employed a semisynthetic approach combining EPL and SPPS to prepare a K^+^ channel containing the D-enantiomer of alanine in place of a conserved glycine residue. X-ray crystallography structures of the semisynthetic protein demonstrated that the ability of the channel to adapt its structure differently for K^+^ and Na^+^ ions is a fundamental aspect of ion selectivity^95^. A detailed discussion of these synthetic endeavours is beyond the scope of this review but readers are referred to a number of excellent recent reviews^11^,^96^,^97^,^98^,^99^. Of particular interest for applications in chemical and molecular biology are proteins displaying defined PTMs such as glycosylation that can be prepared using this approach. The first major example of a glycoprotein prepared through NCL was diptericin, an 82-residue *O*-linked antimicrobial, reported by Bertozzi and co-workers^100^. Since then, the construction of glycopeptides and glycoproteins using NCL has continued with the synthesis of EPO being one of the most impressive examples to date (Fig. 6). EPO is a glycoprotein hormone that stimulates the production of red blood cells and is administered as a therapeutic for anaemia. It represents a substantial synthetic challenge with it comprising of 166 amino acids and bearing four glycosylation sites: three *N*-linked to Asn residues and one *O*-linked to a Ser residue. To further complicate the synthesis, EPO only contains four Cys residues, all of which are at unfortunate positions (7, 29, 33, 161), rendering them unsuitable for NCL. Nevertheless, this glycoprotein was chemically synthesized by Danishefsky and co-workers^101^ using NCL and the desulfurization of Cys to Ala as the key strategy. Kajihara and co-workers have recently reported the synthesis of five homogeneous glycoforms of EPO to probe the biological function of the glycan units^102^. Before this work, Kent and co-workers had reported the chemical synthesis of a homogeneous, polymer-modified erythropoiesis protein (SEP)^103^. This protein construct displayed potent biological activity in cell and animal assays for erythropoiesis and had significantly prolonged *in vivo* half-life. The polymer conjugated EPO derivative reported by Kent is arguably more readily synthetically accessible then the glycosylated versions of Danishefsky/Kajihara and serves to illustrate the power of chemical synthesis for the preparation of improved protein therapeutics.

## Chemical ubiquitination

**Figure 7:**
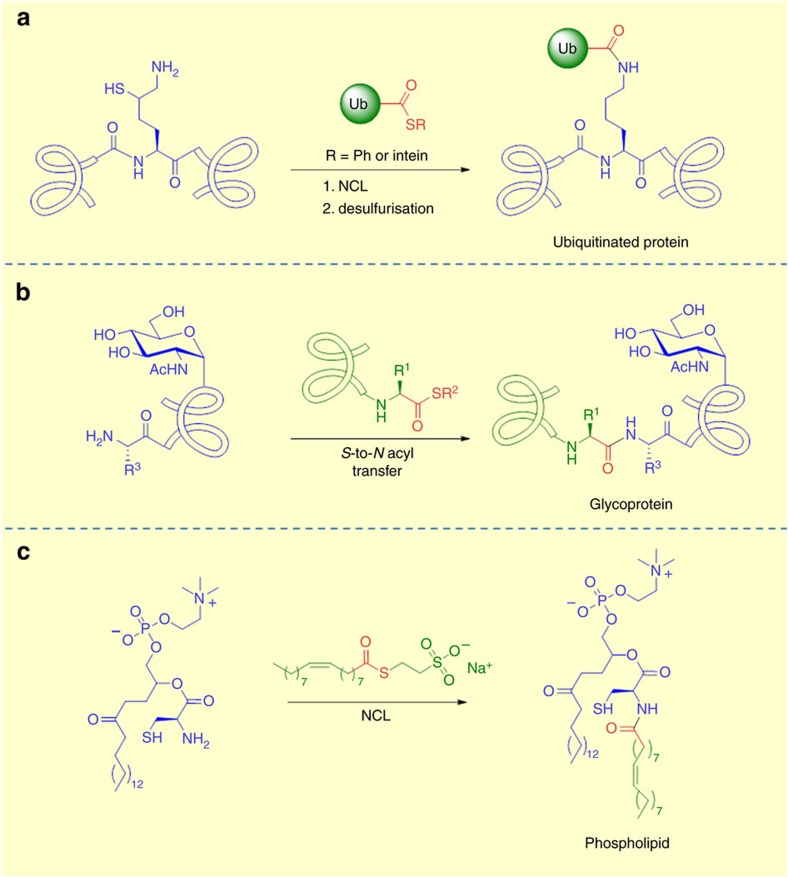
Synthetic applications of S-to-N acyl transfer. (**a**) *S*-to-*N* acyl transfer as a strategy for chemical ubiquitination of proteins (**b**) Intermolecular *S*-to-*N* acyl transfer mediated ligation for the preparation of glycopeptides (**c**) *De novo* synthesis of phospholipids membranes using NCL.

Due to the critical importance of ubiquitination in a range of human diseases, suitable methodologies to prepare practical quantities of homogeneous ubiquitinated proteins are essential to enable detailed biochemical studies and to develop new therapeutics. Methodologies that forge the native isopeptide bond are of particular interest for further biological studies. Both synthetic and enzymatic strategies have been investigated for the preparation of ubiquitinated proteins^31^,^104^,^105^. Although enzymatic approaches are attractive, they require the identification and isolation of the corresponding E2 and E3 ligases associated with ubiquitination of the target proteins, which can be extremely challenging^106^,^107^. Chemical ubiquitination of proteins enables the preparation of homogeneous protein constructs with defined ubiquitination patterns^24^,^108^. Current synthetic methods to introduce native Ub linkages are driven primarily by NCL and EPL approaches^105^,^109^. However, limitations for both of these approaches exist. Cysteine is never found near ubiquitination sites in proteins due to the potential for interference with the enzymatic thioester transfer step, therefore, it must be introduced synthetically or enzymatically for NCL/EPL approaches^110^. Also, formation of the required thioester linkage can be slow and low yielding, in particular for the sterically constrained isopeptide bond of ubiquitination^31^,^111^,^112^. The use of cysteine surrogates such as mercaptolysine has been advanced to overcome the limitations of NCL but these ligations require a chemical desulfurization step to furnish the native bond^59^ (Fig. 7a). Surrogate mediated coupling reactions are also typically slow and are limited to relatively unhindered ligation sites, limiting the scope of the ligation methodology. Recently, Chatterjee and co-workers have reported a highly innovative approach towards native chemical ubiquitination involving the ligation auxiliary 2-aminooxyethanethiol. Following protein ligation through transthioesterification and *S*-to-*N* acyl transfer, the auxiliary is cleaved via homolysis of chemically labile N–O bond to furnish the native Ub linkage. This novel approach was applied to the chemical synthesis of full-length sumoylated histone H4 and histone H2B (ref. 113).

In a seminal study, Muir and co-workers demonstrated that chemical ubiquitination of histone H2B induces direct stimulation of K79-specific methyltransferase hDot1L. Using the photolytic ligation auxiliary developed by the group (Fig. 4), two traceless orthogonal expressed protein ligation (EPL) reactions were used to ubiquitinate H2B site-specifically. Reconstitution of the synthetic uH2B into chemically defined nucleosomes revealed that uH2B directly activates methylation of histone H3 at lysine K79 by methyltransferase hDot1L (ref. 114).

Disulfide-directed ubiquitination has been employed for the site-specific ubiquitination of histone H2B and proliferating cell nuclear antigen (PCNA)^115^,^116^. In this semisynthesis approach, the recombinant Ub-intein conjugate was incubated with cysteamine which undergoes intein-mediated transthioesterifcation followed by *S*-to-*N* acyl transfer to furnish the Ub bearing a C-terminal aminoethanethiol linker. For H2B, the thiol was subsequently activated by reaction with 2,2′-dithiobis(5-nitropyridine), (DTNP) to generate the mixed disulfide reagent. The Ub-thiol derivative and H2B disulfide were allowed to react at pH 6.9 to furnish the disulfide-linked ubiquitylated histone uH2B. In the PCNA system, Ellman's reagent was employed to form the mixed disulfide reagent. Pratt and co-workers^117^ employed disulfide-directed ubiquitination to prepare site-specifically ubiquitin modified α-synuclein derivatives. They demonstrated that different ubiquitination sites have differential effects on α-synuclein aggregation.

In addition to chemical monoubiquitination of proteins, methodologies have also been investigated for the preparation of various poly-Ub substrates^24^,^105^. These strategies involve the chemical ligation of Ub proteins to form polymers before ligation of the poly-Ub onto the protein of interest. One approach that has been investigated for the preparation of Ub polymers is GOPAL (genetically encoded orthogonal protection and activated ligation), by Chin and co-workers^118^. In this approach, the acceptor ubiquitin features a chemically protected Lys residue that is encoded by genetic code expansion. Subsequent orthogonal protection of all other amine groups and chemoselective deprotection of the encoded Lys residue generates an ubiquitin with a single acceptor Lys that is ligated to the thioester of the donor ubiquitin via an *S*-to-*N* acyl transfer, thus forming an isopeptide bond. Deprotection of all remaining amine groups and refolding of the diubiquitin results in native diubiquitin chains. This process is somewhat limited in scope due to the requirement for significant protecting group manipulations. In a seminal study, highlighting the potential of chemical synthesis towards polyubiquitination, Brik and co-workers^119^ prepared α-Syn modified at K12 with poly-Ub (K48 linked chains). This study employed an EPL approach and was synthetically very challenging due to the requirement for desulfurization of the residual thiol at each step. Indeed, this study highlighted some of the potential problems associated with the EPL approach to protein ligation. For example, the attempted ligation reaction between the synthetic Ub-tetramer and α-Syn failed to furnish any of the desired product due to the complete absence of any *trans*-thioesterification. In addition, an attempt to carry out sequential EPL ligation steps followed by a global desulfurization failed to give any of the desired product. The target tet-Ub-α-Syn protein construct was eventually prepared through two sequential ligations of di-Ub with α-Syn. Despite the challenges, this approach highlights the potential of *S*-to-*N* acyl transfer as a key ligation step in protein oligomer synthesis.

## *N*-Glycosylation

Recently, Guo and co-workers have reported an *N*-glycosylation reaction between glycosylamines and *p*-nitrophenyl thioester peptides involving an intermolecular *S*-to-*N* acyl transfer as the key step^120^. The methodology was found to be compatible with fully unprotected glycans. One disadvantage of the methodology is the high reactivity of the thioester, required to promote the intermolecular *S*-to-*N* acyl transfer, renders the process incompatible with aqueous conditions where hydrolysis of the thioester competes with the desired *N*-glycosylation reaction. The intermolecular approach is also limited in terms of chemoselectivity with participating side-reactions from lysine residues. Payne and *et al*.^121^ have reported an intermolecular approach to peptide ligation and glycopeptide synthesis. Hydrolysis of the thioester was minimized through the use of a mixed-solvent system containing *N*-methylpyrrolidinone (NMP) and guanidine hydrochloride (Gn.HCl)/HEPES buffer. The intermolecular process was found to be compatible with all amino acid residues with the exception of lysine that required protection as the lysine(ivDde) group. Kinetic studies comparing the rates of NCL versus the direct intermolecular ligation showed that although NCL is faster than direct aminolysis, intermolecular ligation reactions still proceeded at synthetically useful rates. This finding suggests that some ligation reactions proposed to occur via large-ring transition states, such as SAL, may actually proceed through direct intermolecular aminolysis^121^ (Fig. 7b).

## Synthetic liposomes

Recently, Devaraj and co-workers^122^ have employed an NCL approach to prepare phospholipids spontaneously from thioesters. The chemoselectivity of the intramolecular *S*-to-*N* acyl transfer enables the preparation of amidophospholipids, which self-assemble *in situ* to form membrane bound vesicles. In this system, the NCL process is employed as a non-enzymatic method to drive the self assembly of phospholipid membranes in a manner analogous to the natural lipid synthesis process. The approach was further developed to demonstrate *in situ* formation of phospholipid membranes and concomitant spontaneous reconstitution of functional membrane proteins^123^ (Fig. 7c).

## Applications in supramolecular chemistry and chemical probes

The chemoselective nature of the *S*-to-*N* acyl transfer process and the requirement for mild or neutral reactions conditions renders it an ideal ligation reaction for applications in supramolecular chemistry and for the design of chemical probes.

## Molecular machines

**Figure 8:**
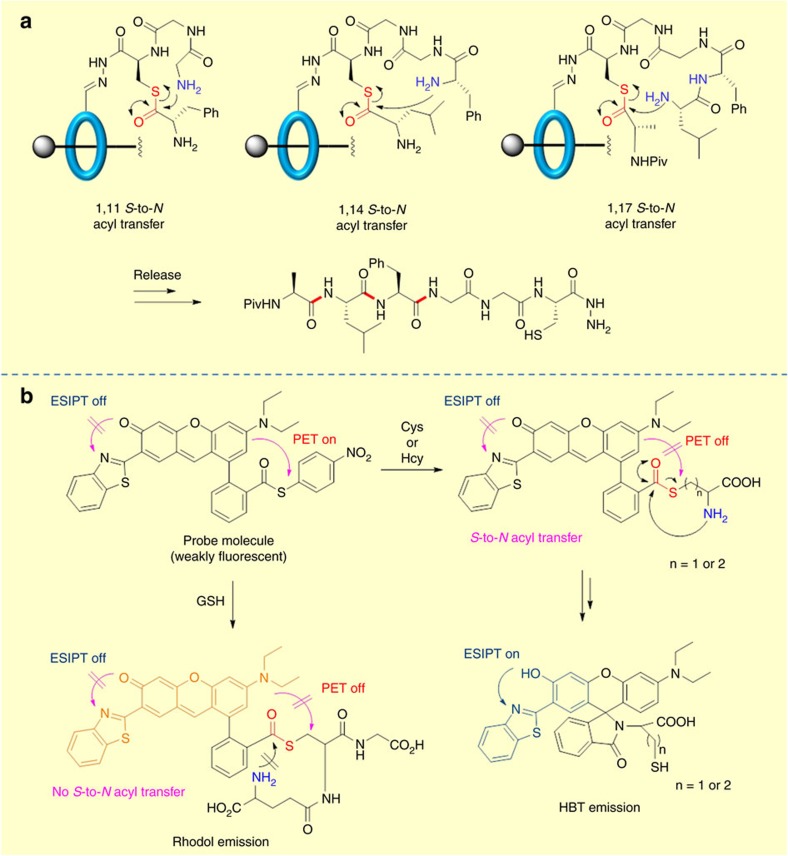
Applications of *S*-to-*N* acyl transfer in supramolecular chemistry and chemical probes. (**a**) Molecular machine functioning through *S*-to-*N* acyl transfer involving increasing cyclic transition state of 11-, 14- and 17-membered rings (**b**) *S*-to-*N* acyl transfer enabled molecular probes.

The ability to control chemoselectivity over large ring sizes lends itself to applications in supramolecular chemistry and in particular to the development of molecular machines. Indeed, with the award of the 2016 Nobel prize in chemistry to Sauvage, Stoddard and Feringa ‘for the design and synthesis of molecular machines' it is anticipated that there will be a surge of interest in reversible chemoselective ligation reactions. The *S*-to-*N* acyl transfer has been employed in a small molecule machine, developed by Leigh and co-workers that mimic ribosomal protein synthesis^124^. A rotaxane ring tethered onto a molecular axel carries a thiolate group that iteratively ligates amino acid residues through an NCL process. The critical peptide bond forming reactions occur through an intramolecular *S*-to-*N* acyl transfer over a gradually increasing cyclic transition state of 11-, 14- and 17-membered rings. The sequential *O*-to-*S*/ *S*-to-*N* acyl transfer/catalyst regeneration/ring movement process highlights the power of sequential chemoselective ligation reactions for the development of sophisticated molecular machines that can mimic complex biological processes (Fig. 8a). Stulz and co-workers^125^ have demonstrated that DNA can be employed to template acyl transfer reactions between thioester modified oligonucleotide and a series of amine and thiol based nucleophiles.

## Chemical probes

The intramolecular *S*-to-*N* acyl transfer offers a chemoselective process that can be applied to the development of sophisticated biological probes suitable for proteomics and imaging. Of particular interest for biological target discovery is the development of Ubiquitin protease probes pioneered by Ploegh and co-workers^126^. Probes for deubiquitinating enzymes (DUBs) involving a native chemical ligation handle have been developed by Ovaa and co-workers^127^. In this approach, native chemical ligation between a Ub thioester and a γ- or δ-thiolysine residue was employed to forge the native isopeptide bond. The subsequent desulfurization step was used to introduce an electrophilic group that can capture the catalytic Cys residue of a DUB. A diubiquitin-based FRET probe that involves an *S*-to-*N* acyl transfer to ligate the FRET pair has also been reported^128^. Strongin and co-workers^129^ have utilized an intramolecular *S*-to-*N* acyl transfer as a key step in the development of a dual emission fluorescent probe that can distinguish between glutathione and cysteine/homocysteine. The probe design includes a reactive thioester moiety that undergoes thioester exchange in the presence of cysteine/homocysteine or glutathione. For cysteine/homocysteine this was followed by an intramolecular *S*-to-*N* acyl transfer over a 5- or 6-membered transition state, triggering a spirocyclization that led to the ‘quinone–phenol' transduction of rhodol dyes, and an excited-state intramolecular proton transfer (ESIPT) process. In the case of glutathione, only the initial transthioesterification took place. This removed the intramolecular photo-induced electron transfer (PET) process caused by the electron deficient 4-nitrobenzene moiety and resulted in a large fluorescence enhancement at the rhodol emission band. No *S*-to-*N* acyl transfer was observed since the transfer would involve a ten-membered cyclic transition state which is disfavoured (Fig. 8b) (a detailed discussion on the size of cyclic transition states in *S*-to-*N* acyl transfer is provided in Box 2). A related strategy was reported by Guo and co-workers using a coumarin hemicyanine fluorescent dye^130^. In this system, only cysteine and no homocysteine or glutathione could participate in the *S*-to-*N* acyl transfer to activate the probe. The authors proposed that for homocysteine, following *trans*-thioesterification, the carboxylate anion could bind more tightly to the benzothiazolium N atom, thereby inhibiting the subsequent intramolecular *S*-to-*N* acyl transfer. These examples highlight the application of *S*-to-*N* acyl transfer in the development of selective chemical probes.

## Outlook and future directions

The unique reactivity of thioester moieties renders them versatile substrates for a wide range of chemoselective processes. Tremendous advancements have been made in exploiting the synthetic potential of both inter- and intramolecular *S*-to-*N* acyl transfer process for a diverse range of applications. The application of small ring-size transition states (<6) remains the most general and robust approach for effecting efficient intramolecular acyl transfer reactions, but applications of larger ring size TS are rapidly becoming more widely utilized. The elucidation of precise, chemoselective acyl transfer reactions in nature serves as inspiration for the development of more sophisticated synthetic acyl transfer processes and in combination with chemoenzymatic/semi-synthesis approaches there appears to be few limitations on the synthetic scope of these processes. For chemical protein synthesis, the ‘cysteine problem' associated with NCL has largely been ameliorated through the development of methods for cysteine-free ligation and desulfurization, however a general approach for traceless protein-protein ligation via NCL remains to be developed. Despite all of the powerful advances in NCL and the wide range of ligation auxiliaries now available, significant challenges still remain in incorporating these handles into full-length proteins, applying them under native folded conditions and subsequently removing them without requiring refolding as the final step. This remains an enormous challenge, which chemical synthesis alone cannot overcome. Therefore, a combination of amber suppression and bioorthogonal chemistry will be required to truly harness the power of chemistry in the semisynthesis of functional proteins. The synthesis of thioesters has been given a considerable boost through advanced synthetic methods and in particular through the introduction of intein-mediated ligation. Nevertheless, the development of novel chemical and enzymatic methods for the formation of thioesters in the presence of fully unprotected complex peptide and protein systems will continue to be an important area of research for the development of the field. The execution of chemoselective *S*-to-*N* acyl transfer reactions across large ring-systems remains a considerable challenge but supramolecular templating approaches and the use of natural/modified ligase enzymes offer intriguing possibilities for site-specificity in these reactions. No doubt, the continued discovery of novel *S*-to-*N* acyl transfer processes in nature and the harnessing of biological machinery will continue to stimulate research in this field. It is clear that the applications of *S*-to-*N* acyl transfer have expanded well beyond the traditional applications of peptide ligation and based on the high chemoselectivity of the process it is anticipated that this trend will continue. The ability to chemically introduce site-specific isopeptide bonds is likely to revolutionize the molecular analysis of biological systems. There is little doubt that the *S*-to-*N* acyl transfer will remain a focus of both chemists and biologists for the foreseeable future.

## Additional information

**How to cite this article:** Burke, H. M. *et al*. Exploring chemoselective *S*-to-*N* acyl transfer reactions in synthesis and chemical biology. *Nat. Commun.* **8**, 15655 doi: 10.1038/ncomms15655 (2017).

**Publisher's note:** Springer Nature remains neutral with regard to jurisdictional claims in published maps and institutional affiliations.

## Reversibility of the *S*-to-*N* acyl transfer.

The equilibrium of the *S*-to-*N*/*N*-to-*S* acyl transfer is thermodynamically favoured towards the amide product and is the principle driving force for amide bond formation^131^. However, the *S*-to-*N* acyl transfer can be pushed into reverse and this process offers a useful methodology for accessing thioesters without relying on the traditional synthetic approaches^41^. In the protein splicing mechanism of inteins, an *N*-to-*S* acyl shift occurs in the first step, and involves transfer of the *C*-terminal amino acid residue of the target protein onto the *N*-terminal side chain -SH of the cysteine residue of the intein^19^. This rearrangement is thermodynamically unfavourable, however, a conformational rearrangement of the intein forces the scissile peptide bond into a high-energy twisted conformation thereby pushing equilibrium towards formation of the thioester^35^. Mutagenesis studies have shown that a highly conserved B-block histidine plays an essential role in splicing by catalysing the *N*-to-*S* acyl transfer^40^. Crystal structures of inteins have shown that the δ1 nitrogen of the B-block histidine is located close to the amide nitrogen of the first residue of the intein, suggesting that the B-block histidine may promote *N*-to-*S* acyl transfer by protonating the leaving group^40^. Several groups have investigated the formation of peptide thioesters based on an initial *N*-to-*S* acyl transfer, mimicking the initial stages of protein splicing^131^. Melnyk and co-workers reported a solid-phase *N*-to-*S* acyl transfer for thioester formation following SPPS using Fmoc/t-Bu chemistry in combination with the sulfonamide safety catch linker^132^. The *N*-to-*S* acyl transfer can proceed with concomitant cleavage from the resin to allow isolation of the thioester. A convenient synthetic approach to promote *N*-to-*S* acyl transfer involves the use of a C-terminal cysteine, α-methylcysteine or a cysteine-proline ester (CPE). C-terminal cysteine mediated transfer is efficient with histidine, glycine or cysteine as amino acids before the cysteine and in the presence of sodium 2-mercaptoethylsulfonate (MesNa)^131^. In these systems, the *S*-to-*N*/*N*-to-*S* acyl transfer equilibrium is displaced towards the thioester form by an intramolecular amide bond forming reaction involving the α-amino group of the Cys residue and an ester group. Melynk and co-workers have described the application of a bis(2-sulfanylethyl)amino (SEA) group on the C terminus with a cysteinyl or homocysteinyl peptide^133^. While working with thiol-auxiliaries for Native Chemical Ligation (NCL), Danishefsky and co-workers reported an *N*-to-*S* acyl transfer driven by protonation of the benzylic amine under acidic auxiliary cleavage conditions^69^. This type of rearrangement was also reported by Vorherr and Aimoto with the 2-mercapto-4,5-dimethoxybenzyl (Dmmb) auxiliary and has been developed into a general method for thioester synthesis^134^,^135^. Nakahara and co-workers described a peptide bearing a C-terminal 5-mercaptomethylated proline derivative that was converted into a thioester on treatment with aqueous 3-mercaptopropionic acid (MPA). The driving force for the initial intramolecular *N*-to-*S* acyl transfer was reasoned as being the lability of the amide bond in the imino acid proline^43^,^136^. It is clear from these and other examples that the equilibrium of the *S*-to-*N*/*N*-to-*S* acyl transfer can be controlled through both biological and chemical approaches resulting in synthetically useful chemical transformations^131^. The reversibility of the *S*-to-*N*/*N*-to-*S* transfer process renders it extremely useful both for complex biological processes and synthetic applications.

## Ring-size in *S*-to-*N* acyl transfer.

*S*-to-*N* acyl transfer reactions in synthesis have been studied across a broad range of ring transition state (TS) sizes, from the widely utilized 5-membered rings found in NCL up to 32-membered cyclic TS^137^. It is widely accepted that a favourable positioning of the amino and thioester moieties in close proximity can enable the selective *S*-to-*N* acyl transfer to occur. This concept is often referred to as ‘entropic activation'^138^. Through a series of detailed mechanistic studies it has been determined that several factors are involved in the outcome of an *S*-to-*N* acyl transfer reaction, these include, ring-size of the TS, conformation, hydrogen-bonding, NH-π interactions, pH, solvent, reactivity/stability of thioester and structure of the AAs in the ring/near the ligation site^137^,^138^,^139^. In addition, the findings of AML have established that templating effects can assist the rate of the intramolecular reaction across a range of ring sizes^75^. This combination of factors can make it extremely challenging to predict the outcome of an *S*-to-*N* acyl transfer involving a macrocyclic TS. Despite these challenges, detailed computational and experimental studies have revealed encouraging trends that allow a degree of predictability to be applied to these processes^137^,^138^. While investigating the synthesis of native peptides from *S*-acyl isopeptides containing a C-terminal cysteine, Katritzky and co-workers studied chemical ligation of *S*-acylated cysteine isopeptides across a range of cyclic transition states. The feasibility of intramolecular acyl migrations via 5- to 19-membered cyclic transition states was demonstrated. Long range *S*-to-*N* acyl transfers were found to depend significantly on the size of the macrocyclic TS, with relative rates, as judged from yields, following the TS ring-size trend, 5>10>11>14, 16, or 17>12>13, 15, or 19>18 ≫9>8 (refs 137, 140). The ratio of the *S*-to-*N* ligation product versus formation of the corresponding intermolecular transacylation product for a 15-membered TS was studied under different pH conditions. The relative rate of ligation versus transacylation (based on product ratio) varied with pH with optimum formation of ligated product recorded at pH 7.0−7.3. Interestingly, the authors found that the chemical ligation proceeding through a16-membered TS appeared to be highly favoured in comparison with the 15-membered TS^141^. Seitz and co-workers investigated internal Cys-mediated ligation reactions proceeding through a macrocylic TS. They found that *S*-to-*N* acyl transfers through 17- to 20-membered macrocylic TS were optimal and that, by contrast, 8-, 11- and 14-membered macrocycles were difficult to form^142^. The *S*-to-*N* acyl transfer in NCL is thermodynamically favourable due to the close proximity of the amino group to the reactive thioester and the involvement of the intramolecular transition state^143^. The first transthioesterification step in NCL is the rate-determining step; however for larger ring sizes (>6 membered ring), the *S*-to-*N* acyl transfer becomes rate-determining step and ring-size of the TS becomes a critical factor in the successful outcome of the process. As in the case of intermolecular *S*-to-*N* acyl transfer, slower formation of the amide bond results in increased competition from thioester hydrolysis. *S*-to-*N* acyl transfer reactions in nature occur over very large ring sizes, often with a high degree of site-specificity. The isopeptide bond forming reactions observed for E3 ligase mediated ubiquitination of proteins occur with incredible precision over an extensive protein-templated macrocycle (Figure 14)^17^. The *S*-to-*N* acyl transfer can be considered to be a proximity-induced process that only occurs when the participating reactive groups are favourably positioned by protein folding. In nature, as in the synthetic examples outlined in this review, there appear to be stringent geometric requirements for successful *S*-to-*N* acyl transfer reactions, the full extent of which remain under investigation.

**Cyclic transition states for** ***S*****-to-*****N*** **acyl transfer reactions.** (**a**) traditional 5-membered TS for NCL (**b**) extended concept of *S*-to-*N* acyl transfer for macrocyclic TS (**c**) specific example of long range *S*-to-*N* acyl transfer (illustrated for 14-membered TS in SAL) (**d**) model for E3 ligase mediated ubiquitination showing acceptor lysine and the thioester linkage (modified from Berndsen and Wolberger^17^).
